# Retractions in Rheumatology: Trends, Causes, and Implications for Research Integrity

**DOI:** 10.1002/acr.80005

**Published:** 2026-02-06

**Authors:** Anna Maria Vettori, Michele Iudici

**Affiliations:** ^1^ International Baccalaureate student, St. Louis School Milan Italy; ^2^ Division of Rheumatology Geneva University Hospitals and University of Geneva Geneva Switzerland

## Abstract

**Objective:**

We aimed to describe the trends and main reasons for study retraction in rheumatology literature.

**Methods:**

We reviewed the Retraction Watch database to identify retracted articles in rheumatology. We recorded the main study characteristics, authors’ countries, reasons for retraction, time from publication to retraction, and trends over time. Reasons for retraction were classified as scientific misconduct, data/figure errors, or other reasons. Main article features and cause of retractions in rheumatology were compared with a sample of articles from other medical specialties.

**Results:**

A total of 381 (79.5% original articles) rheumatology articles were retracted between 1989 and 2024. Most originated from Asia (68.5%), particularly China (50.7%). Scientific misconduct accounted for 75.3% of retractions, followed by data errors (14.9%) and other reasons (7.6%). Common misconduct types included data fabrication, fake peer review, duplication, and authorship issues. The median time from publication to retraction was 18 months (interquartile range 9–46), with one‐third of articles requiring more than 36 months to be retracted. Time to retraction did not improve over time. The number of retractions steadily increased over time from 18 in 2000–2009, 117 in 2010–2019, and 207 in 2020–2023 (*P* < 0.001). Compared with other medical specialties, rheumatology exhibited similar retraction patterns, differing mainly in geographic distribution.

**Conclusion:**

Retractions in rheumatology have risen substantially, largely due to misconduct. This trend may reflect an increase in questionable research practices or improved detection. Strengthening early‐career education, institutional oversight, and ethical research culture is essential to enhance transparency and integrity in the field.

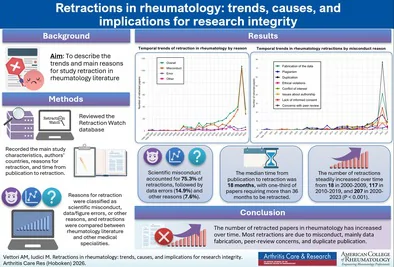

## Graphical abstract

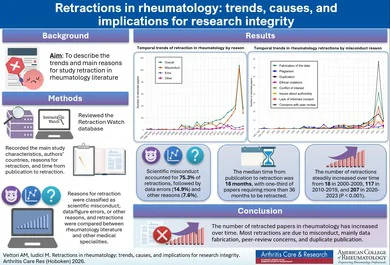

**SIGNIFICANCE & INNOVATIONS**

- The number of retracted articles in rheumatology has increased over time, reflecting either a rise in compromised studies or improved detection.
- Most retractions are due to misconduct, mainly data fabrication, peer‐review concerns, and duplicate publication, whereas only about 15% result from errors without misconduct.
- The findings may help inform medical students, rheumatologists, editors, and institutions, promoting a culture of transparency and accountability in research.

## INTRODUCTION

Scientific publishing plays a central role in the dissemination of medical knowledge and in supporting research and education within the discipline.^1^ However, not all published findings meet the standards of rigor, quality, ethical conduct, and scientific validity. Study validity can be compromised by unintentional errors that naturally occur in research, but in some cases, it may also be undermined by misconduct, including practices such as fraud, plagiarism, or data fabrication.^1^ Ensuring accuracy and credibility in research is essential, as scientific evidence underpins medical decision‐making and health care policy.

Retraction, the formal withdrawal of a published article, is a key mechanism through which the scientific community safeguards research integrity and prevents the dissemination of misleading information that could compromise patient safety, distort clinical practice, or misguide future research.^2^, ^3^ Although still relatively low, the rate of retraction in medicine has risen in recent years, with the frequency and causes of retraction varying considerably across medical fields.^4^, ^5^, ^6^ For instance, the proportion of retractions due to misconduct across medical specialties has been reported to range from 22% to 89%.^6^ When analyzing the reasons for misconduct, plagiarism predominates in some specialties, whereas duplicate publication or data fabrication is more common in others. Similarly, the proportion of articles retracted for unintentional errors is highly variable.^6^ Identifying the patterns and determinants of retractions within specific disciplines is crucial for informing targeted interventions, strengthening editorial and peer‐review practices, and ultimately fostering a culture of research integrity. A specialty‐focused analysis can reveal field‐specific patterns that may be diluted or overlooked in broader biomedical data sets.

To date, no study has specifically examined this issue within the field of rheumatology. We therefore conducted this analysis to characterize retraction patterns in rheumatology, with the aim of understanding their underlying causes and trends, and to identify potential implications for future improvement.

## METHODS

### Search strategy

We performed a review of the Retraction Watch database (RWD) (http://www.retractiondatabase.org) on January 4, 2025. The RWD is continuously updated and provides a comprehensive list of retracted scientific articles. The RWD identifies retractions through automated searches of article databases (eg, PubMed, Web of Science, Scopus), screening of publisher and journal websites, institutional reports of research misconduct investigations, and reader tips. The RWD is updated daily, but some retractions may still be missed or identified with delay, although its coverage generally exceeds that of other sources such as PubMed and Web of Science.^7^ The data set includes, among other fields, the title of the retracted manuscript, the research area (for example, economics or medicine), the institution and country of the corresponding author, the journal, the type of study (for example, clinical research or meta‐analysis), the retraction date, digital identifiers for retraction notices (where available), the DOI of the original article, and the reasons for retraction. To identify articles related to rheumatology, we used a two‐step approach. First, we generated a list of rheumatology‐related terms starting from an initial draft created using a large language model (ChatGPT o3).^8^ The prompt asked the artificial intelligence (AI) to capture different variations of relevant words (for example “rheumatology,” “rheumatism,” etc). For example, to retrieve articles containing terms such as “rheumatology,” “rheumatologic,” “rheumatism,” or “rheumatoid,” the AI suggested using the root “rheuma” as a keyword. It also accounted for language‐specific variations of main disease names. For instance, both “Sjögren” and “Sjogren” were included to capture articles related to Sjögren disease. This process resulted in a list of 65 keywords (Table 1), including the name of the specialty, diseases, major diagnostic tests, treatments, and clinimetric indexes. The list was reviewed for consistency by one of the authors (MI), a rheumatologist. To create the initial shortlist of rheumatology‐related articles, we searched for the presence of at least 1 of the 65 keywords in each row of the Excel file downloaded from retractiondatabase.org. The selected articles were then manually reviewed by the same author (MI) to exclude those not relevant to rheumatology. Discrepancies were resolved by consensus through discussion. To identify medical articles outside the field of rheumatology to use as comparators, we searched the database by filtering the Subject column for articles containing the term “Medicine.” In the RWD, subjects including the word “Medicine” refer to entries within the broader Health Sciences category, which covers a wide range of medical and biomedical specialties such as internal medicine, oncology, cardiology, neurology, surgery, pediatrics, pharmacology, radiology, dentistry, public health, nursing, epidemiology, and related clinical and health‐science disciplines. Articles classified under rheumatology were excluded from the comparator set. We classified reasons for retraction into the following categories, adapted from Resnik et al^9^: scientific misconduct (data fabrication, plagiarism, duplication, ethical violations, conflict of interest, authorship issues, lack of informed consent, compromised peer review, and other/unspecified), errors in data/figure, and other. Information pertaining to the entity initiating or conducting the retraction process (eg, “Investigation by journal/publisher,” “Investigation by institution”) was not considered a substantive reason for retraction. Instead, these cases were grouped separately under the category “Investigation/Administrative,” which records who initiated the retraction but does not represent a specific cause. The impact factors of the journals were assessed using Journal Citation Reports (Clarivate Analytics 2024).^10^ We followed the Strengthening the Reporting of Observational Studies in Epidemiology reporting guideline.^11^

**Table 1:** Keywords used to identify rheumatology articles (alphabetical order)

ANA	gout	rheuma
ankylosing spondylitis	granulomatosis with polyangiitis	rheumatic
anti‐CCP	HAQ	rheumatism
arthritis	health assessment questionnaire	rheumatologic
arthralgia	hyperuricemia	rheumatologist
auto‐inflammatory syndromes	IL‐6 inhibitors	rheumatology
autoimmune disease	immunosuppressants	rheumatoid arthritis
Behcet	inflammation	rheumatoid factor
Behçet's disease	inflammatory arthritis	rheumatoid nodules
biologic DMARD	JAK inhibitors	rituximab
biological therapy	joint degeneration	sarcoidosis
connective tissue disease	joint erosion	scleroderma
corticosteroids	leflunomide	Sjogren
C‐reactive protein	methotrexate	Sjögren's syndrome
CRP	mixed connective tissue disease	spondyloarthropathy
DAS	NSAIDs	spondyloarthritis
DAS28	osteoporosis	sulfasalazine
dermatomyositis	osteoarthritis	synovitis
disease activity score	overlap syndromes	systemic lupus erythematosus
DMARD	PMR	systemic sclerosis
erythrocyte sedimentation rate	polymyalgia rheumatica	TNF inhibitors
ESR	polymyositis	uric acid
fibromyalgia	PsA	vasculitis
GCA	psoriatic arthritis	
giant cell arteritis	remission	

### Statistical analysis

Data were summarized as number (percentage) for qualitative variables and median (interquartile range [IQR]) for continuous variables. Continuous variables were compared with *t*‐test, Wilcoxon test, or Kruskal‐Wallis test, and categorical variables were compared with χ^2^ test or Fisher exact test, as appropriate. We assessed temporal trends using Poisson regression of period counts on an ordinal period index with a log link and an offset for period length (log years); the *P* value is the Wald test for the period coefficient. A *P* value ≤ 0.05 was considered significant. Ethical approval was not required (study not involving human participants). All analyses were performed in R (version 4.4.2).

## RESULTS

### Main study characteristics

Of the retractions retrieved, 381 (published between 1989 and 2024) were related to rheumatology (Figure 1 shows the flowchart of search strategy). Retracted articles in rheumatology were mostly original articles (n = 303; 79.5%), published mainly by authors from Asia (n = 261; 68.5%). China accounted for the largest number of retractions (n = 193; 50.7%), followed by Japan (n = 30; 7.9%). The median number of authors per retracted article was 5 (range 1–46, IQR 3–7), and the median journal impact factor was 3.2 (range 0.2–98.4; IQR 2.5–4.7). In total, articles were retracted from 211 journals, of which 12 had more than five retracted articles (see Supplementary Table S1).

**Figure 1:**
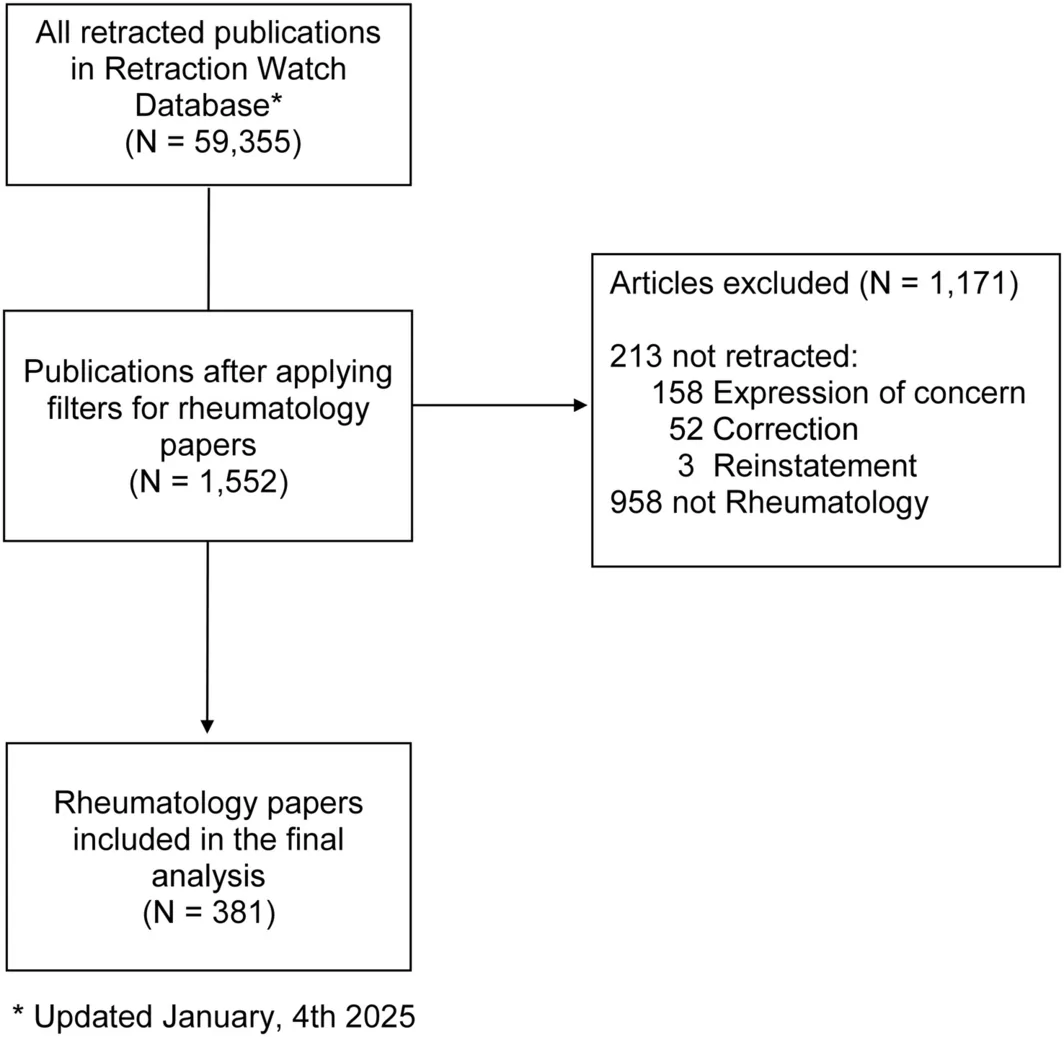
Flowchart of search strategy

### Reasons for retractions

The majority of the articles had multiple reasons for retractions (median 3, IQR 1–4). Most articles had at least one cause of scientific misconduct (n = 287; 75.3%), whereas a smaller proportion of articles were retracted due to data errors without misconduct (n = 57; 14.9%) or other reasons (n = 29; 7.6%). Among the articles retracted for scientific misconduct, the most common issues were data fabrication, concerns with or fake peer review, duplication, and authorship problems (Table 2). Studies retracted for errors without misconduct were published in journals with higher impact factors (median 3.3, IQR 2.8–5.7) than those retracted for misconduct (median 2.9, IQR 2.4–4.3) or for other reasons (median 1.9, IQR 0.8–4.5; *P* < 0.001). Figure 2 and 3 show the temporal trends of retraction in rheumatology by reason and by type of misconduct.

**Table 2:** Main characteristics of retracted articles*

	Rheumatology (n = 381)	Medicine (n = 15,308)	*P* value
Article type			0.056
Original article	303 (79.5)	11,828 (77.3)	
Meta‐analysis/review	50 (13.1)	1,736 (11.3)	
Case report	16 (4.2)	611 (4.0)	
Commentary/editorial/letter	3 (0.8)	224 (1.5)	
Guidelines	1 (0.3)	56 (0.4)	
Other	8 (2.1)	853 (5.6)	
Continent			<0.001
Asia	261 (68.5)	8,923 (58.3)	
Europe	40 (10.5)	2,248 (14.7)	
North America	22 (5.8)	1,720 (11.2)	
Africa	12 (3.1)	349 (2.3)	
South America	6 (1.6)	123 (0.8)	
Oceania	5 (1.3)	152 (1.0)	
Multiple continents	30 (7.9)	1,610 (10.5)	
Unknown	5 (1.3)	182 (1.2)	
Country			<0.001
China	193 (50.7)	5,881 (38.4)	
International	47 (12.3)	2,257 (14.7)	
Japan	30 (7.9)	743 (4.9)	
United States	14 (3.7)	1,509 (9.9)	
India	3 (0.8)	709 (4.6)	
United Kingdom	3 (0.8)	385 (2.5)	
Reason for retraction			0.309
No. studies with ≥1 reason for scientific misconduct	287 (75.3)	11,485 (77.5)	
No. studies retracted for data errors without misconduct	57 (14.9)	1939 (13.1)	
Other reasons	29 (7.6)	1,389 (9.4)	
Scientific misconduct^a^			
Fabrication of the data	96	3,557	
Concern with/fake peer review	94	4,569	
Duplication	87	3,138	
Issues about authorship	67	1938	
Lack or withdrawn of informed consent	62	2,233	
Plagiarism	57	2,243	
Ethical violations	20	962	
Conflict of interest	1	136	
Unspecified misconduct	124	4,458	
Error(s) in the manuscript^a^	267	8,760	
Other reasons^a^	311	12,112	
Journal impact factor, median (IQR)	3.2 (2.5–4.7)	3.1 (2.2–5)	0.976
Time from publication to retraction, months, median (IQR)	18 (9–46)	17 (8–42)	0.584
Retractions due to scientific misconduct	18 (10.5–46)	18 (10–40)	
Retractions for data errors	13 (4–47)	13 (4–34)	
Retractions for other reasons	5 (1–29)	5 (1–25)	
N of authors, median (IQR)	5 (3–7)		
Retraction requesters			0.847
Journals or publishers	140 (48.8)	5,647 (49.3)	
Third party^b^	114 (29.9)	4,392 (28.6)	
Company/institution	33 (8.6)	1,419 (9.3)	
Top 5 publishers			0.244
Hindawi	89 (23.4)	3,643 (23.8)	
Elsevier	42 (11.0)	2012 (13.1)	
Springer	39 (10.2)	1,132 (7.4)	
Wiley	34 (8.9)	1,446 (9.4)	
Taylor and Francis	16 (4.2)	638 (4.2)	
Retraction notice access			
Open access	366 (96.1)	14,754 (96.4)	

* Values are expressed as n (%) unless stated otherwise. IQR, interquartile range.

^a^ More than 1 reason possible for each study retraction.

^b^ An investigation by any third party that is not the United States Office of Research Integrity.

**Figure 2:**
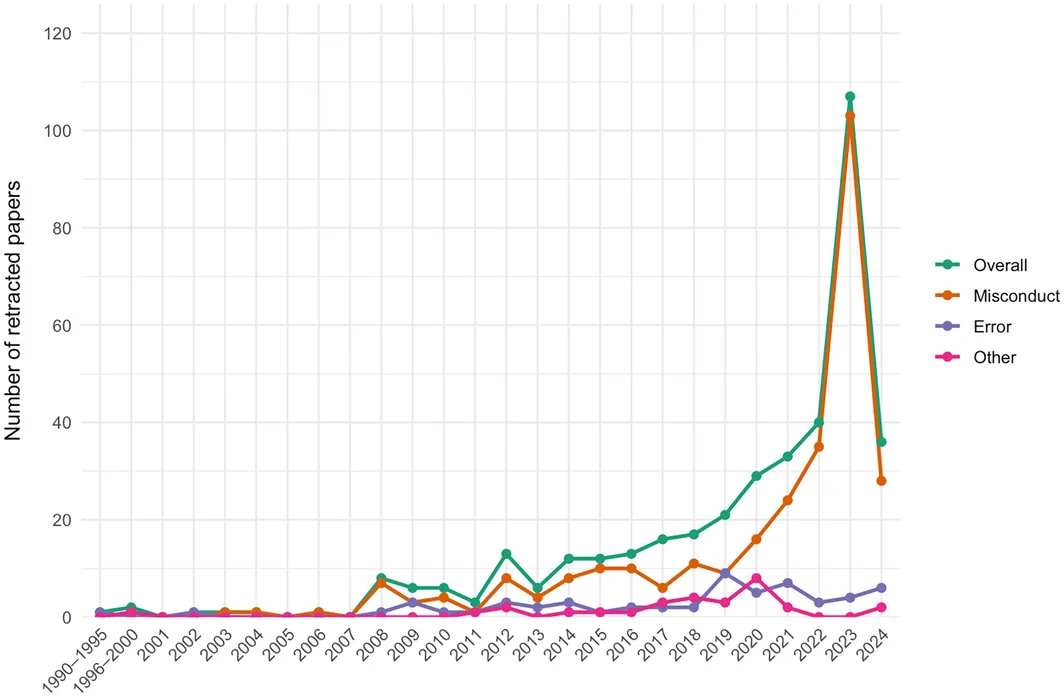
Temporal trends of retraction in rheumatology by reason.

**Figure 3:**
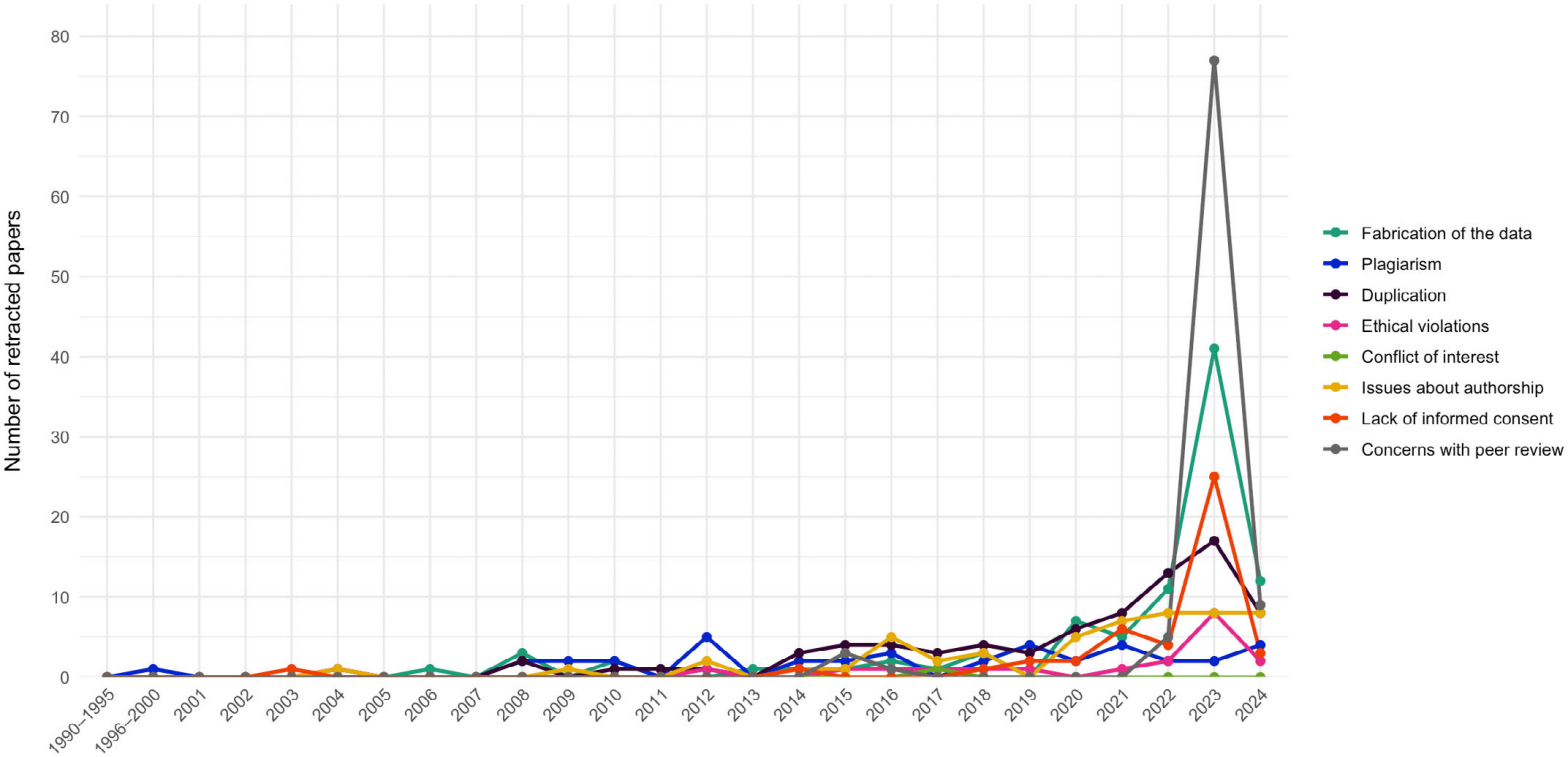
Annual trends in rheumatology retractions by misconduct reason. Color figure can be viewed in the online issue, which is available at http://onlinelibrary.wiley.com/doi/10.1002/acr.80005/abstract.

### Time from publication to retraction

Median time from publication to retraction was 18 months (IQR 9–46, range 1–223), with 118 (31%) and 68 (18%) articles needing more than 36 months and 60 months, respectively, to be retracted. Time needed for retractions was longer for cases of scientific misconduct (18 months, IQR 10.5–46) compared to cases of data errors (13 months, IQR 4–47) or other reasons (5 months, IQR 0–29; *P* < 0.001).

For specific misconduct reasons, time to retraction was shorter for ethical violations, concerns with peer review, lack or withdrawal of informed consent, and plagiarism, whereas it was longer for duplication and authorship issues (see Supplementary Table S2). Time to retraction remained stable over time (median 21 months in 2000–2009, 16 months in 2010–2019, 17 months in 2020–2023; *P* = 0.983).

### Temporal trends of retractions and related reasons

The number of retracted articles increased from 3 in 1990–1999 to 18 in 2000–2009, 117 in 2010–2019, and 207 in 2020–2023 (*P* < 0.001; Figure 2). The year 2023 accounted for the largest number of retractions, with 105 retracted articles (27.5%), of which 101 (96.2%) involved misconduct issues, 92 (87.6%) originated from China, and 74 (70.5%) were published by Hindawi. Similarly, retractions due to misconduct rose from 1 in 1990–1999 to 13 in 2000–2009, 70 in 2010–2019, and 176 in 2020–2023 (*P* for trends < 0.001; Figure 2). Significant upward trends were also observed for retractions due to errors (data not shown), and these trends remained statistically significant even after excluding 2023, the year with a marked spike in retractions.

### Comparison between rheumatology and other retracted studies in medicine

Compared with retracted articles in other medical specialties, retracted rheumatology studies exhibited similar patterns in the reasons for retraction, time from publication to retraction, retraction requesters, and publishers involved. The only significant difference was observed in geographic distribution: a higher proportion of retracted rheumatology studies originated from Asia (68.5% vs 58.3%; *P* < 0.001), particularly from China (50.7% vs 38.4%; *P* < 0.001) (Table 2).

## DISCUSSION

In rheumatology, the number of retractions has steadily increased in recent years, with most cases attributed to scientific misconduct. The leading causes included data fabrication, concerns about peer review, and duplicate publication, whereas only about 15% of retractions resulted from errors without evidence of misconduct. The average time to retraction was approximately 18 months, with no improvement observed over time. Retractions occurred more quickly for cases involving ethical violations, lack or withdrawal of informed consent, peer‐review concerns, and plagiarism, whereas they took longer for issues related to duplication and authorship disputes. Retracted rheumatology studies showed similar patterns to other medical fields, except for a higher proportion originating from Asia, particularly China.

Retractions in rheumatology, as in other medical fields, have increased in recent years. As in other specialties, it is likely that this rise cannot be explained solely by the growing number of publications. In fact, the incidence of retractions has doubled in cardiology^12^ and increased in other fields, from 2.3 per 100,000 publications in 2001 to an estimated 8 per 10,000 publications in 2022.^4^, ^5^ Whether this reflects a general decline in scientific integrity or is instead a consequence of the greater awareness of publication ethics and more effective detection of unreliable work,^13^, ^14^ as advocated by the Committee on Publication Ethics guidelines (2009),^1^ remains uncertain. Nonetheless, it is evident that recent years have seen a stronger emphasis on research integrity, greater public scrutiny through online publication, and wider availability of tools to detect plagiarism or redundant studies. Consequently, the rise in retractions may be interpreted not only as evidence of increased misconduct but also as a reflection of enhanced vigilance and a growing commitment to maintaining scientific standards.^14^ For instance, the spike in article retractions observed in 2023 was largely driven by initiatives from certain publishers to eliminate paper mill content and address cases of compromised peer review.^15^

Regarding the underlying reasons, most retractions were attributed to research misconduct, whereas those resulting from genuine errors, an unavoidable aspect of scientific work that should not be viewed negatively, were a minority. Although variations exist across specialties,^6^ misconduct regrettably remains the predominant cause of retraction in most areas of medicine,^6^, ^16^, ^17^, ^18^, ^19^ with fraud, data fabrication, and fraudulent peer review being the most common forms of misconduct and their incidence showing an increasing trend over time. A meta‐analysis on medical publications found that overall, misconduct was responsible for an estimated proportion of 60% of all retractions, whereas error and publication issues were responsible for, respectively, 17% and 9%.^6^ Several factors may explain why authors engage in unethical research practices. The need to maintain a robust publication record to advance an academic medical career, often described as “publish or perish,” has been widely recognized as a key driver.^20^ Additional contributing factors include inadequate institutional oversight, conflicts of interest, insufficient training in research ethics, and cultural or systemic pressures that prioritize productivity over integrity.^8^, ^14^, ^21^, ^22^ Our findings highlight the need to strengthen education, oversight, and a culture of research integrity in rheumatology, beginning early in training and continuing throughout researchers’ careers. Initiatives such as the EQUATOR Network^23^ and Responsible Conduct of Research programs^24^ endorsed by the National Institutes of Health and the European Commission promote transparency, reproducibility, and ethical research practices. Greater consideration and implementation of such initiatives within rheumatology could further enhance research quality and uphold scientific standards. In this context, the advent of AI presents both a concern and an opportunity: whereas these tools may facilitate image manipulation or the creation of fabricated research content, they also offer the potential for enhanced screening and detection methods that could help prevent such issues.

We did not observe any improvement in the time required to retract an article over the years, which has plateaued at approximately 18 months. Notably, about one‐third of retractions took more than 36 months to be finalized. This highlights the ongoing challenges in identifying flawed articles and taking timely corrective action to minimize their potential negative impact on the scientific literature.^25^ Addressing this issue should be considered a research priority, as recent evidence indicates that retracted articles can distort the results of systematic reviews and meta‐analyses.^25^ Adopting standardized workflows, such as those outlined by the National Information Standards Organization Communication of Retractions, Removals, and Expressions of Concern guidelines,^26^ can streamline decision‐making and ensure prompt correction of the scientific record.

About half of the retracted articles originated from China. In a review of retracted articles across medical fields, China and several low‐ and middle‐income countries showed relatively higher retraction rates compared with their overall publication volume.^27^, ^28^ This pattern may reflect multiple factors, including strong performance‐based incentives in academic promotion systems, varying levels of oversight and mentorship for early‐career researchers,^28^ and the increasing detection of issues such as paper mills or peer‐review manipulation, which may more readily affect regions with rapidly expanding research activity.^29^

Our study has several limitations. First, as mentioned above, we were unable to estimate the exact proportion of retracted publications relative to all publications in rheumatology, given the wide range of diseases encompassed by this specialty, which are difficult to capture within predefined categories. Second, we could not determine whether the rise in retracted publications reflects an absolute increase in erroneous and/or fraudulent research or, rather, heightened detection. Third, we used impact factor values from the 2024 Journal Citation Reports, which may not reflect the journal's impact factor at the time the retracted articles were published. However, this approach is common,^16^, ^30^ and prior studies have shown that citation‐based journal rankings are relatively stable over time, making contemporary impact factors a reasonable proxy for journals’ relative impact.^31^ Fourth, some rheumatology articles, particularly from multidisciplinary journals, may not have been captured, affecting absolute counts. However, this is unlikely to have altered the main conclusions. Finally, it remains uncertain whether these retractions have had a negative impact on subsequent research or patient care. Future research is needed to clarify these points. Strengths of our study include its originality in the field and its systematic approach to characterizing retraction trends, causes, and geographic distribution.

In conclusion, the number of retracted articles in rheumatology has continued to rise over time, primarily due to questionable research practices. This trend may reflect either an increase in the publication of compromised studies or improved detection of such issues. Unfortunately, retractions capture only a small proportion of cases of scientific misconduct. Surveys among scientists have shown that approximately 2% admitted to having fabricated, falsified, or modified data or results at least once, and 34% acknowledged other forms of intentional misconduct, such as manipulating graphs or unjustifiably removing data from final analyses.^22^ Moreover, it is also important to note that cases of misconduct or serious errors are identified during peer review or editorial assessment and therefore never reach publication. These findings highlight the need to raise greater awareness of research integrity issues within the scientific community. Our results may help inform medical students, rheumatologists and trainees, editors, and research institutions, thereby supporting efforts to strengthen preventive strategies, improve editorial and peer‐review processes, and foster a culture of transparency and accountability in research.

## AUTHOR CONTRIBUTIONS

All authors contributed to at least one of the following manuscript preparation roles: conceptualization AND/OR methodology, software, investigation, formal analysis, data curation, visualization, and validation AND drafting or reviewing/editing the final draft. As corresponding author, Dr Iudici confirms that all authors have provided the final approval of the version to be published and takes responsibility for the affirmations regarding article submission (eg, not under consideration by another journal), the integrity of the data presented, and the statements regarding compliance with institutional review board/Declaration of Helsinki requirements.

## Supporting information


**Disclosure form**.

**Table S1:** Number of retracted papers by journal.
**Table S2:** Time from publication to retraction according to the reasons for misconduct.
