# Supplementary material for: Retractions in Rheumatology: Trends, Causes, and Implications for Research Integrity
Source: Arthritis Care Res (Hoboken). 2026 Feb 6;78(10):1372–9. doi: 10.1002/acr.80005 (PMC13613243; doi:10.1002/acr.80005)
Supplement: Supplementary file 1 [file ACR-78-1372-s001.pdf]

Supplementary material

Table S1. Number of retracted papers by journal.

Rank	Journal	N of papers
1	Computational and Mathematical Methods in Medicine	20
2	Evidence-Based Complementary and Alternative Medicine	17
3	Journal of Healthcare Engineering	14
4	BioMed Research International	12
5	Cochrane Database of Systematic Reviews	10
6	Bioscience Reports	6
7	Cureus	6
8	International Immunopharmacology	6
9	Annals of the Rheumatic Diseases	5
10	Clinical Rheumatology	5
11	PLoS One	5
12	Rheumatology International	5
13	Arthritis and Rheumatism	4
14	Biomedicine & Pharmacotherapy	4
15	Contrast Media & Molecular Imaging	4
16	Disease Markers	4
17	Expert Opinion on Pharmacotherapy	4
18	Molecular Medicine Reports	4
19	Yonsei Medical Journal	4
20	Cell Cycle	3
21	Emergency Medicine International	3
22	European Review for Medical and Pharmacological Sciences	3
23	Inflammation	3
24	Journal of Cellular Physiology	3
25	Journal of Environmental and Public Health	3
26	Journal of Orthopaedic Surgery and Research	3
27	Life Sciences	3

Rank	Journal	N of papers
28	Oxidative Medicine and Cellular Longevity	3
29	Rheumatology	3
30	Scientific Reports	3
31	The Journal of Immunology : Official Journal of the American Association of Immunologists	3
32	A New Day in Medicine (Новый День В Медицине)	2
33	Aging	2
34	Arthritis & Rheumatology	2
35	Arthritis Research and Therapy	2
36	Calcified Tissue International	2
37	Cell Biochemistry and Biophysics	2
38	European Spine Journal	2
39	Experimental and Therapeutic Medicine	2
40	Frontiers in Immunology	2
41	Inorganic and Nano-Metal Chemistry	2
42	International Journal of Molecular Medicine	2
43	Journal of Advanced Nursing	2
44	Journal of Biological Regulators and Homeostatic Agents	2
45	Journal of Clinical Laboratory Analysis	2
46	Lupus	2
47	Medical Science Monitor: International Medical Journal of Experimental and Clinical Research	2
48	Medicine	2
49	Molecular Biology Reports	2
50	Osteoporosis International	2
51	PNAS: Proceedings of the National Academy of Sciences of the United States of America	2
52	RSC Advances	2
53	Romanian Journal of Internal Medicine	2
54	The Journal of Rheumatology	2
55	World Journal of Orthopedics	2

Rank	Journal	N of papers
56	2011 5th International Conference on Bioinformatics and Biomedical Engineering	1
57	Acta Histochemica	1
58	Advanced Healthcare Materials	1
59	Advances in Materials Science and Engineering	1
60	American Journal of Kidney Disease	1
61	American Journal of Nephrology	1
62	American Journal of Respiratory Cell and Molecular Biology	1
63	American Journal of Translational Research	1
64	Applied Nanoscience	1
65	Arabian Journal of Chemistry	1
66	Archives of Dermatological Research	1
67	Archives of Gynecology and Obstetrics	1
68	Archives of Osteoporosis	1
69	Archives of Pharmacal Research	1
70	Artificial Cells, Nanomedicine, and Biotechnology	1
71	Asian Pacific Journal of Tropical Medicine	1
72	Autoimmune Diseases	1
73	Autoimmunity	1
74	Autoimmunity Reviews	1
75	BMC Complementary Medicine and Therapies	1
76	BMC Medical Imaging	1
77	BMC Musculoskeletal Disorders	1
78	BMC Pharmacology and Toxicology	1
79	BMC Rheumatology	1
80	BMJ: British Medical Journal	1
81	Best Practice & Research. Clinical Rheumatology	1
82	Biochemical Genetics	1
83	Biochemistry and Cell Biology: Biochimie et Biologie Cellulaire	1
84	Bioengineered	1

Rank	Journal	N of papers
85	Biomarkers in Medicine	1
86	Blood	1
87	Bone	1
88	Case Reports in Rheumatology	1
89	Cell Death & Disease	1
90	Cell Transplantation	1
91	Clinical Anatomy	1
92	Clinical Immunology : the Official Journal of the Clinical Immunology Society	1
93	Clinical Orthopaedics and Related Research	1
94	Clinical Rehabilitation	1
95	Clinical and Translational Medicine	1
96	Current Drug Safety	1
97	Current Pharmaceutical Design	1
98	Cytokine	1
99	Der Orthopäde	1
100	Diagnostic Pathology	1
101	Dose-Response	1
102	Draft Evidence Report: Janus Kinase Inhibitors for Rheumatoid Arthritis	1
103	Drug Delivery and Translational Research	1
104	Drug Design, Development and Therapy	1
105	Egyptian Journal of Chest Diseases and Tuberculosis	1
106	Egyptian Rheumatology and Rehabilitation	1
107	European Heart Journal Cardiovascular Imaging (formerly known as European Journal of Echocardiography)	1
108	European Journal of Clinical Nutrition	1
109	European Journal of Medical Research	1
110	European Journal of Radiology Open	1
111	Experimental Cell Research	1
112	FEBS Open Bio	1
113	Frontiers in Cardiovascular Medicine	1

Rank	Journal	N of papers
114	Frontiers in Genetics	1
115	Frontiers in Medicine	1
116	Fukushima Journal of Medical Science	1
117	Future Generation Computer Systems	1
118	Gastroenterology Research and Practice	1
119	Genetic Testing and Molecular Biomarkers	1
120	Georgian Medical News	1
121	Health and Quality of Life Outcomes	1
122	Heart, Lung and Circulation	1
123	Human Gene Therapy	1
124	Human Genetics	1
125	IJC Heart & Vasculature	1
126	Immunity, Inflammation and Disease	1
127	Immunopharmacology and Immunotoxicology	1
128	Inflammation Research	1
129	Internal Medicine	1
130	International Journal of Applied and Fundamental Research (Международный Журнал Прикладных И Фундаментальных Исследований)	1
131	International Journal of Clinical and Experimental Medicine	1
132	International Journal of Clinical and Experimental Pathology	1
133	International Journal of Gynecology & Obstetrics	1
134	International Journal of Molecular Sciences	1
135	Joint Bone Spine	1
136	Journal of Applied Biomaterials & Functional Materials	1
137	Journal of Arthritis	1
138	Journal of Autoimmune Diseases	1
139	Journal of Bone and Mineral Research: the Official Journal of the American Society for Bone and Mineral Research	1
140	Journal of Cardiology Cases	1
141	Journal of Cellular Biochemistry	1

Rank	Journal	N of papers
142	Journal of Cellular and Molecular Medicine (JCMM)	1
143	Journal of Clinical Rheumatology: Practical Reports on Rheumatic & Musculoskeletal Diseases	1
144	Journal of Clinical and Diagnostic Research (JCDR)	1
145	Journal of Cluster Science	1
146	Journal of Dermatology & Dermatologic Surgery	1
147	Journal of Experimental Medicine	1
148	Journal of Fundamental and Applied Sciences	1
149	Journal of Hand Surgery (European Volume)	1
150	Journal of Health Psychology	1
151	Journal of Inflammation Research	1
152	Journal of International Medical Research	1
153	Journal of Medical Ultrasound	1
154	Journal of Molecular Structure	1
155	Journal of Nanomaterials	1
156	Journal of Obstetrics and Gynaecology	1
157	Journal of Obstetrics and Gynaecology Canada	1
158	Journal of Oral & Facial Pain and Headache	1
159	Journal of Orthopaedic Science: Official Journal of the Japanese Orthopaedic Association	1
160	Journal of Osteoporosis and Physical Activity	1
161	Journal of Patient-Reported Outcomes	1
162	Journal of Physics: Conference Series	1
163	Journal of Prosthodontic Research	1
164	Journal of Sensors	1
165	Journal of Spinal Disorders and Techniques	1
166	Journal of Translational Medicine	1
167	Korean Journal of Pain	1
168	La Presse Médicale	1
169	Lancet	1
170	Modern Rheumatology	1

Rank	Journal	N of papers
171	Molecular Medicine	1
172	Molecular Therapy - Nucleic Acids	1
173	Molecular and Cellular Biochemistry	1
174	Musculoskeletal Disease Associated with Diabetes Mellitus	1
175	Nanomaterials	1
176	Nature	1
177	Nature Immunology	1
178	Nature Medicine	1
179	Naunyn Schmiedeberg's Archives of Pharmacology	1
180	Nephrology	1
181	Neurology	1
182	Neuropeptides	1
183	Oncotarget	1
184	Otolaryngology - Head and Neck Surgery	1
185	PLoS Genetics	1
186	Parkinsonism & Related Disorders	1
187	Pharmacology	1
188	Phytotherapy Research (PTR)	1
189	Procedia - Social and Behavioral Sciences	1
190	Reumatismo	1
191	Reumatologia	1
192	Reumatología Clínica	1
193	Revista Cubana de Medicina Militar	1
194	Rheumatology Reports	1
195	Romanian Journal of Morphology and Embryology	1
196	Russian Medical and Biological Bulletin Named After Academic I.P. Pavlova (Российский Медико-Биологический Вестник Имени Академика И.П. Павлова)	1
197	Scandinavian Journal of Rheumatology	1
198	Scanning	1
199	Scientific Programming	1

Rank	Journal	N of papers
200	Sechenov Medical Journal (Сеченовский вестник)	1
201	Seminars in Arthritis and Rheumatism	1
202	South African Journal of Psychology	1
203	The American Journal of Medicine	1
204	The Journal of Clinical Investigation (JCI)	1
205	The Journal of International Medical Research	1
206	The Journal of Pain : Official Journal of the American Pain Society	1
207	Therapeutics and Clinical Risk Management	1
208	Traditional Medicine Research	1
209	Trials	1
210	Wireless Communications and Mobile Computing	1
211	Wound repair and regeneration: Official Publication of the Wound Healing Society and the European Tissue Repair Society	1

Table S2. Time from publication to retraction according to the reasons for misconduct.

Scientific misconduct	Months from publication to retraction, median (IQR)
Fabrication of the data	19.2 (12.9 - 50.7)
Concern with/Fake peer review	13.9 (11.2 - 18.0)
Duplication	46.1 (15.4 - 72.2)
Issues about authorship	27.2 (7.82 - 58.6)
Lack or withdrawn of informed consent	14.6 (10.6 - 21.0)
Plagiarism	16.1 (7.08 - 46.9)
Ethical violations	13.6 (9.4 - 35.8)
Unspecified misconduct	20.7 (14.4 - 53.2)

For Peer Review Only
